# Paracelsus in nanotoxicology

**DOI:** 10.1186/s12989-014-0035-7

**Published:** 2014-08-12

**Authors:** Dominique Lison, Giulia Vietti, Sybille van den Brule

**Affiliations:** 1 Louvain Centre for Toxicology and Applied Pharmacology, Brussels, Belgium

## Abstract

No abstract.

## 

Nanotoxicology came to light in the years 2000, and rapidly became The Eldorado in toxicology, attracting many investigators, notably because significant public funding was available to address the growing health concerns posed by nanomaterials (NM) and their technological applications. Almost 10,000 publications dealing with the toxicity of NM are currently referenced in PubMed, and citation metrics reflect an enthusiastic activity of the nanotoxicology community. The most highly cited papers in toxicology journals are almost exclusively dealing with NM, e.g. among the 10 most cited publications in *Particle and Fibre Toxicology* identified in May 2014, nine are about NM. This blooming enthusiasm for nanotoxicology has been a source of inspiration for many scientists, leading to innovative methodological developments, new findings and discoveries and we are currently beginning to discern how NM interact with cells, biological tissues and their components. It is, however, fair to acknowledge that nanotoxicology has not yet delivered the results expected by regulators and public authorities. Voices have even questioned the usefulness and scientific validity of many nanotoxicology data produced in recent years [1]. Recommendations have been made to improve nanotoxicology methods and the quality of research [2]. A first step was addressed by improving NM characterization. The necessity to carefully assess, describe and report the physicochemical characteristics of the NM investigated experimentally has been acknowledged early on [3],[4] and most editorial boards will currently not accept publishing a manuscript that does not include a decent characterization of the test materials. The challenge for nanotoxicologists is now to use this physicochemical information to decipher the complex mechanisms of nanoparticle toxicity.

Another source of debate is related to the definition and expression of the dose in nanotoxicology. After 500 years, the famous dictum of Paraceslus “*Dosis Sola Facit Venenum”* is still a key concept in modern toxicology, but it has not been easily integrated in the field of nanotoxicology. In this commentary, we focus on the very basic but complex aspects of dose and dosimetry in *in vitro* nanotoxicology. In the following section, we describe and define important terms and concepts related to this issue.

*Exposure* is generally defined as a condition entailing a possibility for an agent to enter in contact with a biological target, an organism, a tissue or a cell. The *dose* is defined as the amount of agent which enters in contact with the biological target (as a consequence of exposure). A *metric* is a standard of measurement, a way of expressing exposure or dose. Many metrics have been proposed in nanotoxicology, including μg/ml, cm^2^/ml, μg/cm^2^, number particles/ml, … for *in vitro* measurements. Dosimetry is the accurate measurement of the dose.

An accurate definition of the dose is needed to identify and characterize dose-effect and dose–response relationships that contribute to characterize the hazard of a chemical. It can, in principle, be expected that the closer to the effect the dose is defined, the more accurate will be the dose-effect/response relationships. In conventional toxicology, it is, therefore, usually preferred to use biologically effective (delivered, cell-associated or intracellular) doses compared to nominal dose or exposure.

In particle toxicology, and notably in nanotoxicology, we are dealing with solid materials that may have different modes of interaction with the cells, and defining the biologically effective dose is not a trivial issue (Figure 1). In nanotoxicology, the dose is defined by default as the nominal dose, i.e. the amount of NM introduced into the culture medium, but the possibility exists to also measure deposited, cell-associated or intracellular doses. Delivered or cellular doses in nanotoxicology are conditioned by the very specific behavior of nanoparticles in biological media, notably in cell culture medium. Their movements and deposition in biological fluids are determined by different forces including diffusion, gravitation and convection, the relative importance of which varying with the particle shape, surface charge, size and/or density as well as with the composition of the medium [5]. Nanoparticles tend to agglomerate and aggregate to reduce their surface energy and bind biomolecules that modify their surface characteristics. As a consequence, investigators have rapidly realized that NM are significantly modified, both qualitatively and quantitatively, before entering in contact with the cells at the bottom of the culture well. Qualitative modifications are addressed by investigating surface adsorption of biomolecules from the culture medium, leading to the formation of the so-called (protein) corona [6],[7]. Efforts are now directed at characterizing the NM in the test medium, not only before the experiment but possibly also *in operando* (during or at the end of the experiment) [8]. In addition, cultured cells produce biomolecules that may interact with NM and possibly modify their behavior in suspension [9]. Quantitative aspects are addressed by several investigators who realized the need for a closer attention to the dosimetry of nanoparticles *in vitro*. This led to the development of the “particokinetics” concept [10], and studies trying to delineate the biologically effective dose of nanoparticles [5],[11].

**Figure 1:**
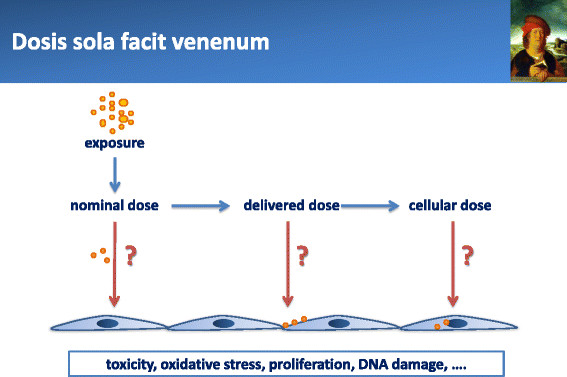
**Biologically effective doses in** ***in vitro*** **nanotoxicology.**

The matter remains, however, tricky and there is probably no one-fits-all approach to address dosimetric questions in nanotoxicology. We highlight here the complexity of these issues in an effort to guide the selection of most appropriate dose metrics, according to the research question, the NM investigated and the endpoint of toxicity considered.

1. **Considering delivered and cellular doses.**

Investigators should now systematically consider the possibility that the nominal dose might not be sufficiently accurate to characterize dose-effect/response relationships, and should examine the dose delivered to the cells, or the (intra)cellular doses. The delivered dose can be estimated by modelling [12],[13] and some researchers have started reporting dose-effect relationships with these parameters instead of the conventional nominal dose [14],[15]. It should also be considered that the cellular uptake of NM is an active process that can be influenced by the type and activation state of the cultured cells. The uptake of nanoparticles by cultured J774 macrophages is e.g. increased after priming with LPS [14]. Cell density is another parameter to consider as it will obviously affect the cellular dose, leading to reduced cytotoxicity at higher cell density [16]. The cell-associated or intracellular doses can often be determined by quantifying the NM or a representative component, and a range of methods are available in function of the NM considered, including chemical analysis, fluorescence, luminescence, radioactivity or spectroscopy.

2. **But, the cellular dose is not always the most relevant.**

The biologically effective dose might in some cases be extracellular like for zinc oxide nanoparticles which mainly exert their cytotoxic activity through the release of Zn(II) cations [17]. More complex mechanisms have, however, been reported for other metallic nanoparticles such as copper [18] and cobalt oxide [19], involving the cellular uptake of the nanoparticles and the subsequent intracellular release of toxic cations (Trojan horse mechanism). Based on this mechanistic knowledge, one might wish to preferably rely on the nominal dose for ZnO nanoparticles (reflecting extracellular Zn(II) ions), and on the intracellular dose for CoO and CuO nanoparticles. Other NM act by interacting with the cell membrane and some specific receptors [20], suggesting that cell uptake is not critical for mediating the toxic effect, hence the deposited or cell-associated dose should be considered as the most relevant. On other occasions, the toxicity of nanoparticles might be mediated by their capacity to modify the culture medium, e.g. by depleting it from essential nutrients [21] or by activating some biomolecules [22], which might again suggest that the nominal dose would be more appropriate to account for the cytotoxic activity. Knowledge of the mechanism of toxicity, obtained experimentally or deduced by analogy with a similar NM, is therefore essential to guide the investigators selecting the most relevant dose to characterize dose-effect/response relationships.

3. **Disperse or not?**

Most investigators in nanotoxicology take great care to adequately disperse the nanoparticles in culture medium by adding chemical dispersants and/or using mechanical dispersion such as sonication. This practice should be examined critically on a case by case basis to determine whether NM really need to be dispersed before cell exposure and whether this is relevant for real exposure and/or effects under study. It is indeed well-known that nanoparticles in the air, in water or in food are largely aggregated/agglomerated, and the relevance of exposing cells to well-dispersed suspensions that do not occur in the “real life” might be questioned. One might also wish to ask whether the cellular response is actually affected by the dispersion state. This issue remains an apparent puzzle because some authors found that the formation of aggregates reduces the cytotoxic activity of NM, whereas others did not observe an influence of the aggregation/agglomeration state. The heterogeneity of these findings might, in part, be explained by considering that some studies used chemicals to disperse the nanoparticles in suspensions [23] and hence concomitantly modified their surface characteristics (see below). The influence of the aggregation state may also vary with the cellular endpoint examined. We have e.g. reported that the cytotoxic activity of nanosilica particles towards macrophages was not affected by their degree of aggregation [24] whereas their membranolytic activity in erythrocytes decreased with the degree of aggregation [25]. It should also be considered that dispersants (tension-active agents, proteins) act by modifying the surface properties of the nanoparticles and hence modify their surface reactivity; it might therefore be difficult to determine whether a response change recorded after adding a dispersant is due to the dispersion state or to surface modification. High energy sonication can also produce free radicals that initiate a variety of chemical reactions in the culture medium and might modify the NM or be a confounding source of cytotoxicity [26]. Hence, we should keep in mind that changing one NM parameter may cause a chain reaction leading to the modification of several other parameters. Given the complexity of the dispersion issue, no single recipe can be recommended and investigators should strive to rationally justify if and how to disperse their NM.

4. **Which metric to express the dose?**

Expression of the dose in nanotoxicology remains another difficult issue as there is no agreement on the most appropriate approach, probably reflecting the fact that different situations require different approaches. However, the idea that “effects of NM are not related to the mass dose” is not correct. Most investigators report the gravimetric dose (μg/ml culture medium, μg/cm^2^ culture well, μg/10^6^ cells, …), and this is probably a reasonable initial choice to verify that a cellular response to a given NM is a dose-dependent phenomenon. For a given NM, a dose-effect/response relationship can often be evidenced with the mass dose, even if the most relevant metric is particle surface area or number because those metrics are inter-related. A confounding effect may, however, arise when comparing several NM and it might be appropriate to consider another metric than mass to better compare samples. For instance, when it appears that cytotoxicity is driven by the release of reactive oxygen species, this is generally a surface-driven phenomenon for insoluble NM and the results should be normalized for the specific surface area. The surface area dose is, in contrast, probably not appropriate for NM exerting their toxicity through the release of soluble ions (see above ZnO, CuO, CoO) and mass dose should be preferred in this case (although solubility might be a function of surface area).

How to express the dose of high aspect ratio NM (HARN) such as carbon nanotubes is even more conjectural. By analogy with what we know from the conventional toxicology of fibres, it would appear sensible that the most relevant metric to characterize the dose-effect/response relationships for HARN is the number of “fibres”. There is, however, no consensus on what is a “carbon nanotube fibre”: single nanotubes, ropes or aggregates of nanotubes, long nanotubes, long ropes? This is a very difficult question that will need to be adequately addressed as it directly impacts regulatory aspects such as the definition of an occupational exposure limit (OEL) for carbon nanotubes.

For every complex problem there is an answer that is clear, simple, and wrong H.L. Mencken

Thus, we have accumulated experience and knowledge in nanotoxicology and the core assumption of Paracelsus remains robustly valid in the field. Designing, performing and mainly interpreting a nanotoxicology experiment *in vitro* is, however, a complex venture that requires controlling many parameters. We should not be under the illusion that simple and univocal recipes exist, and the reliable implementation of high throughput systems for evaluating the health hazard of NM should be expected in the long-term rather than in the near future. Investigators should recognize this complexity and integrate the need for critically adapting implemented protocols and metrics according to the research question, the type of NM investigated, and, possibly, analogies based on knowledge about mechanisms of action.
